# Performance of plant-produced RBDs as SARS-CoV-2 diagnostic reagents: a tale of two plant platforms

**DOI:** 10.3389/fpls.2023.1325162

**Published:** 2024-01-04

**Authors:** Mattia Santoni, Noemi Gutierrez-Valdes, Denise Pivotto, Elena Zanichelli, Anthony Rosa, Guillermo Sobrino-Mengual, Juliette Balieu, Patrice Lerouge, Muriel Bardor, Riccardo Cecchetto, Monica Compri, Annarita Mazzariol, Anneli Ritala, Linda Avesani

**Affiliations:** ^1^ Diamante SB srl, Verona, Italy; ^2^ VTT Technical Research Centre of Finland Ltd., Espoo, Finland; ^3^ Department of Biotechnology, University of Verona, Verona, Italy; ^4^ Université de Rouen Normandie, Normandie Univ, GlycoMEV UR 4358, SFR Normandie Végétal FED 4277, Innovation Chimie Carnot, IRIB, GDR CNRS Chemobiologie, RMT BESTIM, Rouen, France; ^5^ Applied Plant Biotechnology Group, Department of Plant Production and Forestry Science, University of Lleida-Agrotecnio CERCA Center, Lleida, Spain; ^6^ Department of Diagnostics and Public Health, Microbiology Section, University of Verona, Verona, Italy; ^7^ Azienda Ospedaliera Universitaria, UOC Microbiologia e Virologia, Verona, Italy

**Keywords:** COVID-19 pandemic, RBD production, serological tests, diagnostics, plant-based biologics, transient expression, BY-2 cell culture, glycan profiles

## Abstract

The COVID-19 pandemic has underscored the need for rapid and cost-effective diagnostic tools. Serological tests, particularly those measuring antibodies targeting the receptor-binding domain (RBD) of the virus, play a pivotal role in tracking infection dynamics and vaccine effectiveness. In this study, we aimed to develop a simple enzyme-linked immunosorbent assay (ELISA) for measuring RBD-specific antibodies, comparing two plant-based platforms for diagnostic reagent production. We chose to retain RBD in the endoplasmic reticulum (ER) to prevent potential immunoreactivity issues associated with plant-specific glycans. We produced ER-retained RBD in two plant systems: a stable transformation of BY-2 plant cell culture (BY2-RBD) and a transient transformation in *Nicotiana benthamiana* using the MagnICON system (NB-RBD). Both systems demonstrated their suitability, with varying yields and production timelines. The plant-made proteins revealed unexpected differences in N-glycan profiles, with BY2-RBD displaying oligo-mannosidic N-glycans and NB-RBD exhibiting a more complex glycan profile. This difference may be attributed to higher recombinant protein synthesis in the *N. benthamiana* system, potentially overloading the ER retention signal, causing some proteins to traffic to the Golgi apparatus. When used as diagnostic reagents in ELISA, BY2-RBD outperformed NB-RBD in terms of sensitivity, specificity, and correlation with a commercial kit. This discrepancy may be due to the distinct glycan profiles, as complex glycans on NB-RBD may impact immunoreactivity. In conclusion, our study highlights the potential of plant-based systems for rapid diagnostic reagent production during emergencies. However, transient expression systems, while offering shorter timelines, introduce higher heterogeneity in recombinant protein forms, necessitating careful consideration in serological test development.

## Introduction

The COVID-19 pandemic prompted scientists around the world to search for solutions against the SARS-CoV-2 coronavirus. This has opened several collaborations even over disciplines, speeded up developments in science, and allowed for an agile progression of technologies in fighting a rapidly evolving global health problem. A sufficient production of SARS-CoV-2-related recombinant proteins has been crucial for their applications in different frameworks, e.g., diagnostic, active immunization, and protection. The plant community, as part of the joint effort to help combat COVID-19, has also contributed through many initiatives (Capell et al., 2020; Diego-Martin et al., 2020; Tusé et al., 2020; Lobato Gómez et al., 2021), providing further evidence that plants are efficient platforms for the expression of complex and glycosylated viral proteins.

Plant-based recombinant COVID-19 works have mostly been based on transient expression in *Nicotiana benthamiana* (see **Table 1** and references therein). Only few studies involved the stable transformation of plant cell suspensions (Daniell et al., 2022; Rebelo et al., 2022; Ferreira et al., 2023; Shen et al., 2023). Among the proteins reported, there are structural proteins such as the spike (S) protein or S subunits (S1 and S2), the receptor-binding domain (RBD), the nucleocapsid (NC), envelope (E), and membrane (M) viral proteins, and others such as angiotensin-converting enzyme 2 (ACE2) receptor and antibodies against domains of the spike protein (RBD and S1) and the nucleocapsid protein (**Table 1**). The first plant-made COVID-19 vaccine, Covifenz^®^, is a relevant example of plant-based biologics, as it has been approved for human use by Health Canada (Government of Canada, 2022). This vaccine, manufactured by Medicago Inc., is produced in *N. benthamiana* by transient expression and consists of recombinant spike glycoproteins as virus-like-particles (VLPs) with an adjuvant. Covifenz^®^ has represented a great success story for the plant community to advance more plant-based biologicals. While the approval of a plant-made VLP-based COVID-19 vaccine was a huge success for the field, the manufacturing was discontinued in 2023 based on management decisions in the light of changes in the global COVID-19 vaccine demand and market environment. Despite this recent blowback, all these milestones demonstrate the huge potential of plants as bioreactors for biopharmaceutical production (Benvenuto et al., 2023; Eidenberger et al., 2023).

**Table 1:** Published plant-manufactured COVID-19-related proteins (March 2020–August 2023).

Stable transformation				
Protein	Species		Protein accumulation level	Reference
**RBD and S protein**	*Nicotiana tabacum* BY-2 and *Medicago trucantula*	Cell suspension	n/a	(Rebelo et al., 2022)
**RBD**	*Medicago trucantula*	Cell suspension	0.5–2.8 mg/L	(Ferreira et al., 2023)
**ACE-2**	*Lactuca sativa*	Transplastomic plants	17 mg/g DW	(Daniell et al., 2022)
**Anti-SARS-CoV-2 antibodies**	*Nicotiana tabacum* cv Wisconsin	Cell suspension and transgenic plants	16.6 ± 2.3 mg/mL	(Shen et al., 2023)
Transient expression				
Protein	Species		Protein accumulation level	Reference
**S protein**	*Nicotiana benthamiana*	Leaves	n/a	(Ward et al., 2021)
			n/a	(Margolin et al., 2022)
			8–23 mg/kg FW^a^	(Jung et al., 2022)
**E, M, and NC proteins**			200 mg/kg FW^a^	(Williams et al., 2021)
			n/a	(Moon et al., 2022)
			22 mg/kg FW^a^	(Mamedov et al., 2021b)
**S1 domain**			n/a	(Makatsa et al., 2021)
**Rbd**			25 mg/kg FW^a^	(Siriwattananon et al., 2021b; Jirarojwattana et al., 2023)
			1–10 mg/kg FW^a^	(Shin et al., 2021)
			20 mg/kg FW^a^	(Schwestka et al., 2021)
			n/a	(Royal et al., 2021)
			8 mg/kg FW^a^	(Rattanapisit et al., 2020)
			20 mg/kg FW^a^	(Rattanapisit et al., 2021)
			100 mg/kg FW^a^	(Mardanova et al., 2021)
			20–90 mg/kg FW^a^	(Mardanova et al., 2022a)
			20 mg/kg FW^a^	(Mardanova et al., 2022b)
			20–22 mg/kg FW^a^	(Mamedov et al., 2021c)
			6 mg/kg FW^a^	(Maharjan et al., 2021)
			n/a	(Makatsa et al., 2021)
			n/a	(König-Beihammer et al., 2022)
			20–28 mg/kg FW^a^	(Khorattanakulchai et al., 2022)
			2–4 mg/kg FW^a^	(Diego-Martin et al., 2020)
			30 mg/kg FW^a^	(Ceballo et al., 2022)
			n/a	(Yun et al., 2023)
			42–45 mg/kg FW^a^	(Mamedov et al., 2023)
**Anti-SARS-CoV-2 antibodies**			4–35 mg/kg FW^a^	(Shanmugaraj et al., 2020)
			130 mg/kg FW^a^	(Rattanapisit et al., 2020)
			n/a	(Kallolimath et al., 2021)
			131 mg/g FW^a^	(Jugler et al., 2022)
			n/a	(Jaewjaroenwattana et al., 2022)
			35–150 mg/kg FW^a^	(Frigerio et al., 2022)
			73–192 mg/g FW^a^	(Diego-Martin et al., 2020)
**ace-2**			100 mg/kg FW^a^	(Siriwattananon et al., 2021a)
			400–500 mg/kg FW^a^	(Mamedov et al., 2021a)
			50 mg/kg FW^a^	(Castilho et al., 2021)
			91 mg/kg FW^a^	(Jirarojwattana et al., 2023)
			50 mg/kg FW^a^	(Izadi et al., 2023)

S, spike protein; S1 and S2, S subunits; RBD, receptor-binding domain; NC, nucleocapsid protein; E, envelope protein; M, membrane protein; ACE2, angiotensin-converting enzyme 2.

a The reported accumulation levels are converted when possible to milligrams per kilogram fresh weight to facilitate comparison.

n/a, not available.

Although transient expression, as a production platform, allows a fast response to urgent protein demands, it also requires continuous agroinfiltration of plant leaves, maintenance of an abundant number of plants, and large greenhouse facilities. An alternative protein production platform is the use of cell suspension cultures, such as BY-2, which are aseptic and homogeneous and allow scale-up in a controlled closed environment. These cultivation conditions make plant cell suspensions easily adaptable systems for Good Manufacturing Practices compliance. Elelyso^®^, a pharmaceutical for the treatment of type 1 Gaucher disease and the first plant-made biologic approved, is, for instance, produced in carrot cell cultures (Shaaltiel et al., 2007; Ratner, 2010; Walsh, 2014).

In the framework of the recent pandemic, different quantitative tests have been developed to measure SARS-CoV-2 antibodies and study sero-prevalence in the population, with relevant importance to characterize the extent of infection- or vaccine-induced immunity. For this purpose, mainly nucleocapsid (NC)- and spike protein (S)-based assays have been used in serological tests to measure the antibody titers in sera with different outcomes in terms of diagnostic performance (Favresse et al., 2021). While antibodies against NC are strongly induced early on in infected individuals (Le Bert et al., 2020), their physiological significance is unclear. The antibodies directed toward S-protein are likely to be neutralizing (Wajnberg et al., 2020), with different studies that show a correlation between spike protein binding assays and various forms of functional virus neutralization (Bonelli et al., 2020; Padoan et al., 2020; Patel et al., 2021). However, it has been shown that only RBD-specific antibodies demonstrate excellent correlation with neutralization assays already in the early phase of infection (Klausberger et al., 2021).

In this work, we evaluated the feasibility of developing a reliable serological test based on RBD with plant-made reagents and assessed differences in the performance of the reagents produced via transient expression or stable transformation. For this, we manufactured functional recombinant RBD proteins in two distinct plant platforms: *N. benthamiana* leaves and *Nicotiana tabacum* BY-2 cell suspension. We compared the structure of the two plant-made proteins, their post-translational modifications and manufacturability for an ideal test setup. Finally, we developed two quantitative, enzyme-linked immunosorbent assay (ELISA)-based serotests relying on the SARS-CoV-2-receptor-binding domain (RBD) antigens made in plants and compared the results obtained with the commercial serological test mainly used in clinical practice.

## Materials and methods

### Generation of plant expression vectors

**Figure 1:**
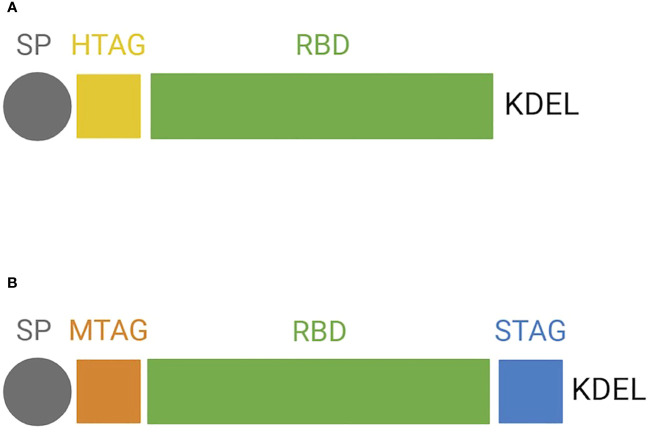
Protein structure of the receptor-binding domain (RBD) forms expressed in plants. Schematic representation of **(A)** RBD expressed transiently in *N. benthamiana* and **(B)** RBD expressed stably in BY-2. SP, signal peptide; HTAG, 6× histidine tag; KDEL, tetrapeptide sequence for ER retention; MTAG, cMyc tag; STAG, Strep tag.

For *N. benthamiana* transient expression, the DNA sequence encoding the RBD (aa 319-541; Amanat et al., 2020) portion of the SARS-CoV-2 S protein (GB: MN908947.3) was modified by codon optimization for plant host and by adding at the N-terminus a plant signal peptide (SP; Pogrenbyak et al., 2005), followed by 6× histidine tag and at the C-terminus the endoplasmic reticulum (ER) retention signal (KDEL) (**Figure 1A**).

The gene obtained was then introduced in the 3′ TMV-based vector pICH31070 as previously reported by Avesani et al. (2014), yielding pICH31070.RBDKDEL, which was inserted into *Agrobacterium tumefaciens* GV3101.

For BY-2 stable expression of RBD, two different vectors were generated: one for its co-expression with ER-targeted GFP (pNGV010) and one without GFP (pNGV011). The use of GFP was meant as a visual aid for the culture selection of BY-2 cell lines that are shown to hold a high level of heterogenicity and may require a long-term subculturing regime to result in homogeneous lines (Häkkinen et al., 2018). Codon-optimized RBD, harboring at the N-terminus the same SP described above, was cloned into pCAMterX (Joensuu et al., 2010). In both pNGV010 and pNGV011, RBD was modified by adding c-myc tag at the N-terminus and Strep II tag and a KDEL signal at the C-terminus (**Figure 1B**). The vectors were transformed into *A. tumefaciens* EHA101.

### *In planta* RBD expression

*N. benthamiana* plants were grown from seed and cultivated at 25°C with 40–60% relative humidity and a light/dark cycle of 16/8 h. *A. tumefaciens* cells carrying pICH20111, pICH14011, and pICH31070.RBDKDEL were grown and used for syringe infiltration of three leaves of 4- to 5-week-old *N. benthamiana* plants as previously described (Gecchele et al., 2015; Santoni et al., 2019).

For BY-2, the cells were transformed as previously described (De Sutter et al., 2005). Transgenic BY-2 cell lines were kept as calli on modified MS medium (Nagata and Kumagai, 1999) with 1% agar and 25 mg/L kanamycin and subcultured every 3 to 4 weeks by visual selection of the most fluorescent biomass under UV light (for the GFP-encoding cell lines) or by selecting actively growing callus on the selective antibiotic plates. Suspension cultures were cultured in liquid (50 mL) of the modified MS medium supplemented with 50 mg/L kanamycin and subcultured weekly by transferring 5% (v/v) of the culture to fresh media.

A selected transgenic BY-2 line expressing RBD was cultivated in a 20-L batch in Biostat C (Sartorius AG, Goettingen, Germany) by inoculating at 5% (v/v) with a 7-day-old culture. The modified MS medium (Nagata and Kumagai, 1999) was prepared and sterilized in the bioreactor before cultivation. The cultivation was carried out in dark conditions at 28°C for 5 days. Conditions such as CO_2_%, pH, %DO, FW, and DW were monitored over the cultivation period. Pack cell volume was measured by sampling 10 mL of suspension culture in a conical tube and centrifuging at 3,220 × *g* for 10 min. The total biomass was collected with vacuum filtering using a Larox filter press (PF 0.1 H2) and Aino T30 filter cloths, applying ca. 3–5 bar pressure, weighed, and freeze-dried at −56°C for 72 h in a CHRIST ALPHA1‐4 LD plus chamber before the preparation of the soluble crude extract.

### RBD detection

For *N. benthamiana* material, total soluble proteins (TSP) were extracted from the plant matrix by grinding the tissue with liquid nitrogen. The powder was then re-suspended in three volumes of extraction buffer (1× phosphate-buffered saline [PBS], 0.1% Tween-20) supplemented with cOmplete™ EDTA-free protease inhibitor (COEDTAF-RO). The homogenate was centrifuged at 30,000 × *g* for 20 min at 4°C.

For BY-2 material, TSP extraction was performed with freeze-dried biomass disrupted using steel beads and a Retsch mill (MM301, Haan, Germany). The powder was then disrupted and homogenized with 1× phosphate-buffered saline, 12 mM Na_2_HPO_4_·2H_2_O, 3 mM NaH_2_PO_4_·H_2_O, 150 mM NaCl, 1 mM EDTA, 100 mM sodium ascorbate, and 1.12 mL/L protease inhibitor cocktail (P9599, Sigma Aldrich, St. Louis, MO), and centrifuged at 3,220 × *g* for 10 min at RT.

The protein concentration of both TSP extracts, from *N. benthamiana* and BY-2, was determined using the Bradford reagent (Sigma B6916).

The presence of RBD in the homogenate was detected by Western blot analysis. Briefly, equal quantities of TSP were loaded onto a 12% reducing SDS-PAGE (SurePAGE). After electrophoretic separation, the proteins were transferred onto a nitrocellulose membrane by electroblotting, incubated with anti-RBD antibodies (Thermo Fischer PA5 -11691), and diluted 1:2,000.

For detection of RBD from *N. benthamiana*, an anti-rabbit antibody (Thermo Fischer 31460) conjugated with horseradish peroxidase and diluted 1:10,000 was used as secondary antibody and ECL™ Select Western Blotting Detection Reagent as detection system (Amersham), followed by signal capturing by Chemidoc™ (BioRad).

For detection of RBD from BY-2, a goat anti-rabbit IgG (H+L) secondary antibody DyLight 680 (35568, Thermo Fisher) diluted 1:10,000 was used, and Odyssey DLS Imaging System (Li-cor Biosciences, USA) was employed as the detection system. The positive control used is described in each Western blot.

For BY-2, c-myc and Strep II tags were detected via Western blotting; the same membrane was revealed twice with different antibodies. To detect c-myc, the blot was incubated with the c-Myc primary antibody (A00172, Genscript) diluted 1:1,000, followed by IRdye 680RD Goat anti-Rabbit IgG (926-68071, Li-cor) diluted 1:25,000 and Odyssey DLs Imaging System (Li-cor Biosciences, USA) as the detection system. On the next day, the same blot was rinsed and incubated for at least 2 h with the Strep-Tactin AP conjugate secondary antibody (2-1503-001, IBA Lifesciences) diluted 1:2,000, followed by BCIP/NBT substrate (S3771, Promega), and Odyssey DLs Imaging System (Li-cor Biosciences, USA) was used for detection.

### RBD purification from *N. benthamiana*

RBD purification from *N. benthamiana* harvested material has been carried out with a modified version of the protocol described in Bortesi et al. (2009). Briefly, 10 g of leaf tissue was homogenized in four volumes of 5 mM imidazole, 10 mM ascorbic acid, and 0.1% PBS-T, pH 8, and centrifuged at 15,000 × *g*, 4°C, for 15 min. The supernatant was passed on a filter paper. A pre-purification step, as described in Santoni et al. (2022), was performed using a DEAE column. The flow-through was collected, adjusted at 500 mM NaCl, and filtered through a 0.2-µm filter. The RBD was then purified with a Hi-TRAP column (Cytiva). The purity of the protein preparation was analyzed by loading aliquots of the eluted fractions on reducing SDS-PAGE stained with Coomassie (proteinArk Serva Biotech). The RBD-containing fractions were pooled and dialyzed against PBS, pH 8, yielding purified NB-RBD.

### RBD purification from BY2

The BY-2-produced RBD was purified using Strep tag-based purification. Following TSP extraction as described above, the sample was diluted 1:10 with 1× cold PBS buffer and passed through a 0.45-μm PES filter. From this point onward, the sample was consistently kept cold on ice. After preparing and equilibrating the AKTA system, the diluted extract was transferred onto a StrepTrap HP column (29048653, Cytiva). Any unbound and nonspecific proteins attached to the column were washed out using 10 column volumes (CV) of 1× PBS at pH 7.4. Elution was carried out by using 6 CVs of 2.5 mM desthiobiotin in PBS. The purified samples were adjusted to pH 8, yielding purified BY2-RBD.

### Protein quantification

The purified proteins were quantified with bicinchoninic acid assay (Pierce BCA ThermoFischer) and by densitometry analysis on SDS-PAGE stained with Coomassie using a calibration curve made with BSA from 0 to 1.25 µg. The results were analyzed using ImageLab software (Bio-Rad).

### Analytical size exclusion chromatography

The purified RBDs, NB-RBD and BY2-RBD, were subjected to size exclusion chromatography using a fast protein liquid chromatography (FPLC) system, model AKTAstart (Cytiva, Milan, Italy). The purified protein was loaded on the gel filtration column Sephacryl Hiprep S-100 HR (Cytiva, Milan, Italy), equilibrated, and eluted with PBS pH 8. Then, 4-mL fractions were collected, and absorbance at 280 nm was recorded.

### Glycosylation analysis

Affinodetection with concanavalin A (Con A) and immunodetections with antibodies raised against core β(1,2)-xylose (anti-xylose antibodies, Agrisera) and α(1,3)-fucose (anti-fucose antibodies, Agrisera) were performed according to the literature (Bardor, 1999). The *N*-glycan profiles of NB-RBD and BY2-RBD were determined through analysis by LC-ESI MS/MS of peptides and glycopeptides released by trypsin and Glu-C digestions of purified RBDs as previously described in Balieu et al. (2022) for glycoproteomic and glycan profiling of the SARS-CoV-2 S protein produced in *N. benthamiana*.

### RBD IgG ELISA

Indirect ELISA has been carried out as reported by Klausberger et al. (2021) and Shin et al. (2021). Plant-made RBDs were diluted to 6 ng/µL in PBS, pH 7.4, and 50 µL was dispensed in Maxisorp polystyrene 96-well plates (ThermoFischer). After overnight incubation at 4°C, the plates were washed three times with 200 µL/well of PBS + 0.1% Tween-20 (v/v) (PBS-T) and blocked with 3% milk (w/v) dissolved in 0.1% PBS-T for 1 h at room temperature (RT). Furthermore, 1% milk in PBS-T was used to dilute the serum samples to 1:200, and 100 µL was dispensed in every well and incubated for 2 h at RT. After three steps of washing with 200 µL PBS-T 0.1%, 100 µL of anti-human IgG conjugated with AP, diluted to 1:10,000 in 1% milk PBS-T 0.1% (Sigma A9544), was dispensed in every well and incubated for 1 h at RT. Three consecutive washes with PBS-T 0.1% and one with PBS were performed, followed by 100 µL of pNPP (chementek) and 30 min of incubation at RT in the dark. The reaction was blocked with 0.1 M NaOH, and the absorbance values were read at 405 nm. To calculate the sensitivity and specificity of the test, two different thresholds were set: the former calculated as the average of healthy sera plus three times their standard deviation and the latter as the average of healthy sera plus twice their standard deviation.

To quantify the antibody titer in each serum, a calibration curve was set up as described in Klausberger et al. (2021) using monoclonal anti-RBD antibody (CR3022, Absolute Antibody) at different concentrations expressed in U/mL, where U = 100 ng of anti-CR3022, generating a curve from 0 to 125 U/mL.

### Euroimmun serological test

The Euroimmun anti SARS-CoV-2 ELISA IgG, based on the recombinantly expressed spike protein domain S1, was carried out to determine the immune response in serum samples following the manufacturer’s instructions. The results were assessed semi-quantitatively by the calculation of the ratio of the extinction of the samples and that of the calibrator. The ratio was interpreted as follows: <0.8—negative, ≥0.8 to <1.1—borderline, and ≥1.1—positive. Serial dilution analyses were performed to provide a semiquantitative result when the OD samples exceeded the OD system limit.

### Participants

In total, 20 serum samples from in-patients and three serum pre-COVID19 samples from in-patients were collected. Based on the current regulation on spared serum samples from the diagnostic workup, considering the venous blood sampling as part of the routine clinical practice and the observational nature of the study carried out without any action on the patients themselves, a formal approval by the ethical committee or a signed informed consent was not required.

### Statistics

Statistical analyses were performed in Prism (Graphpad Prism 9.0; Graphpad Software Inc., San Diego, CA, USA). The absorbance value of every serum was measured in a technical triplicate, and the sera with variation coefficients higher than 5% were discarded. Pearson’s correlation coefficients were used for the serological method comparison.

## Results

### RBD production in *N. benthamiana* leaves

**Figure 2:**
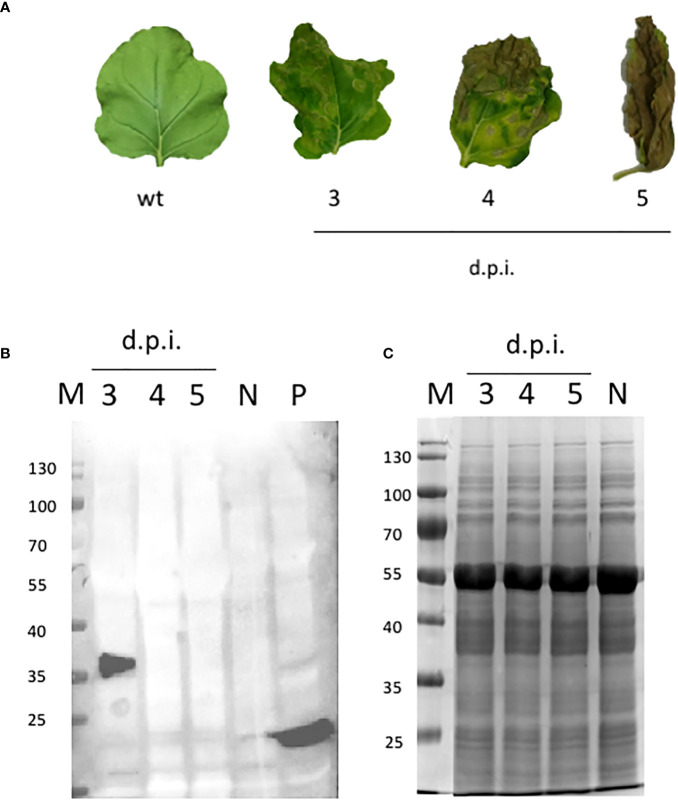
NB-RBD time-course expression analysis. **(A)** Leaves detached from *N. benthamiana* agro-infiltrated leaves either with GV3101 *A tumefaciens* (N) or expressing receptor-binding domain (RBD) at different days post-infiltration 3–5 (d.p.i.). **(B)** Western blot analysis of the same plant samples using the polyclonal anti-RBD PA5-114529 antibody. **(C)** SDS-page Coomassie staining. N, negative control (leaves infiltrated with *A tumefaciens* GV3101); P, positive control (100 ng of a cytosolic RBD produced in *E coli*); M, molecular marker.

*N. benthamiana* was manually infiltrated with MagnICON-vector modules, pICH31070.RBDKDEL- (bearing a modified RBD sequence for ER retention, **Figure 1A**), pICH20111-, and pICH14011, and sampled 3 dpi. After 3 dpi, the plants showed necrosis symptoms (**Figure 2A**), suggesting RBD toxicity, as previously reported by Diego-Martin et al. (2020). Highly necrotic leaves harvested at dpi 4 and 5 did not express RBD as illustrated by Western blot, performed on equal amounts of TSP (**Figure 2C**), using an anti-RBD antibody (**Figure 2B**).

**Figure 3:**
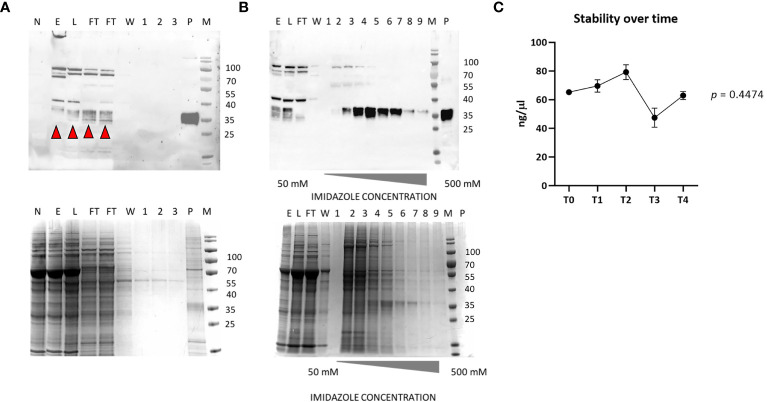
NB-receptor-binding domain (RBD) purification. **(A)** Western blot of anion exchange chromatography (top) and its SDS-PAGE Coomassie stain (bottom). **(B)** Western blot of affinity chromatography using the polyclonal anti-RBD PA5-114529 antibody (top) and its SDS-PAGE Coomassie stain (bottom). **(C)** RBD stability over-time from T0 to T4 (6 months). M, molecular marker; P, positive control (100 ng of a commercial RBD produced in HEK293, purity 90%); E, crude extract; L, load; FT, flow-through; W, wash; 1–3, elution fractions of DEAE; 1–9, elution with imidazole-raising concentrations. The red arrows indicate the monomeric RBD form.

**Figure 4:**
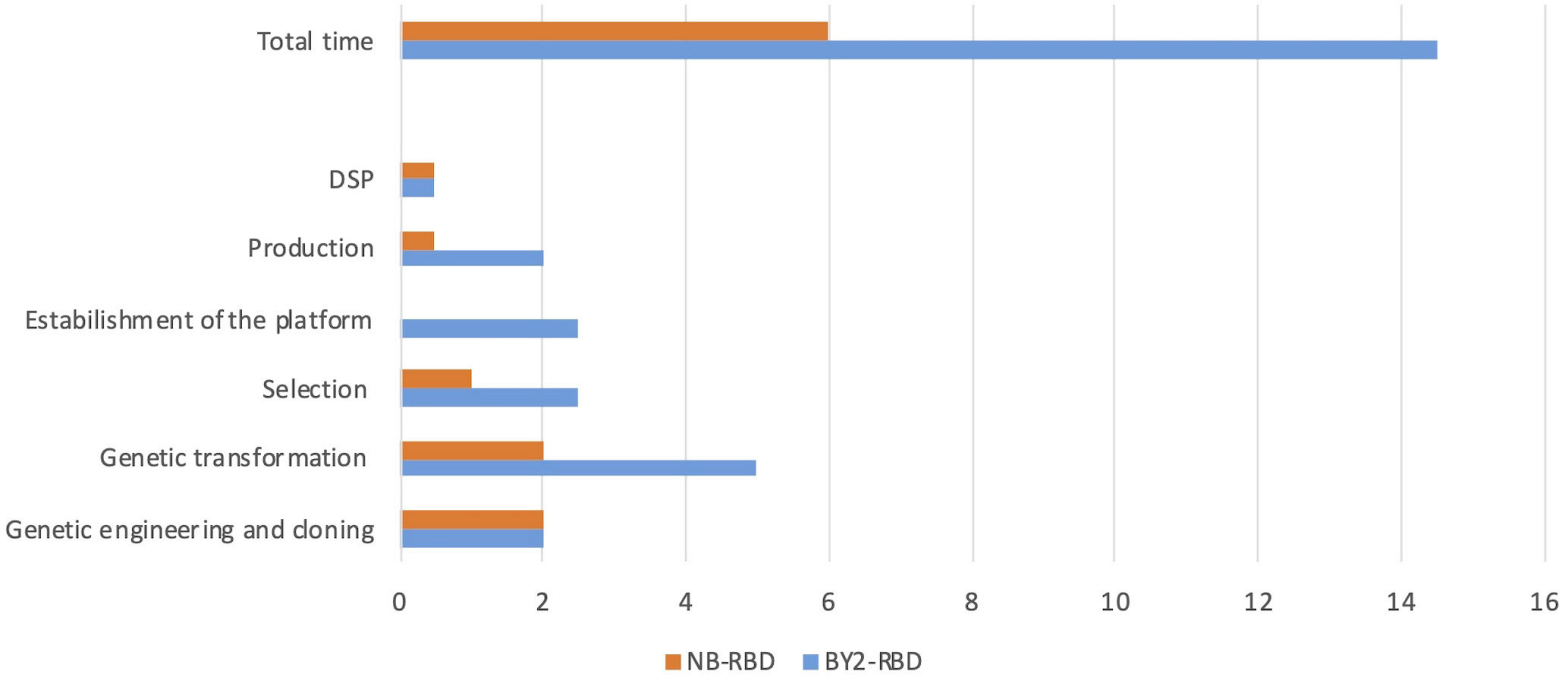
NB-receptor-binding domain (RBD) and BY2-RBD production phases and timelines. In the x-axis, the weeks requested to reach each milestone indicated in the y-axis are reported. DSP, downstream processing.

The accumulation level of RBD in *N. benthamiana* leaves when collected at 3 dpi showed average values of 73 µg/g ± 10 of leaf fresh weight (LFW). Small-scale purification of NB-RBD was performed by anion exchange and affinity chromatography (**Figures 3A, B**) with average yields of 19 µg/g ± 5 of LFW, which is between two- and fivefold higher than what is reported in literature (Diego-Martin et al., 2020; Klausberger et al., 2021). The average time required to manufacture NB-RBD was estimated to be 6 weeks. **Figure 4** reports the total timeframe broken down into specific experimental activities.

The purified NB-RBD is stable if stored at -80°C in PBS-T 0.1%, pH 8, for over 6 months (**Figure 3C**).

### RBD production in BY-2 cells

**Figure 5:**
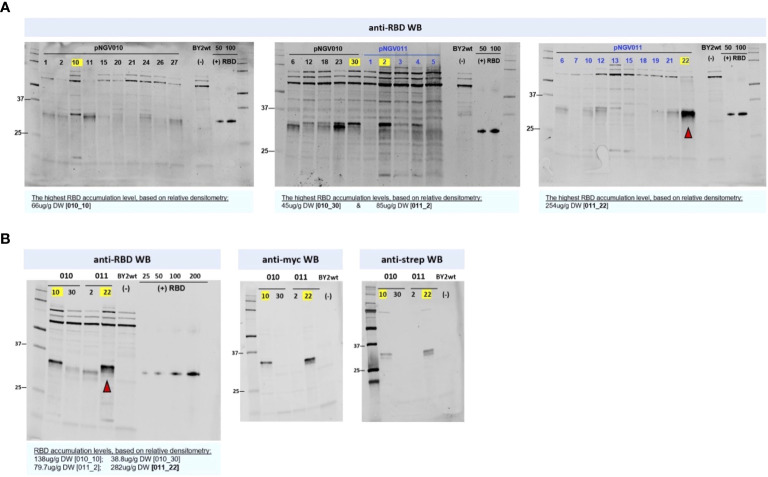
Receptor-binding domain (RBD) screening in BY2 clones. Equal quantities of soluble crude extract were loaded into each lane. Soluble crude extract from wild-type BY-2 was used as negative control for all blots. The yellow labels mark the highest expressor clones per construct in each blot. **(A)** The pNGV010 set is portraying RBD in co-expression with ER-targeted GFP, and the pVNG011 set is portraying RBD without GFP. The highest estimated RBD accumulation for the latter (22) is indicated with a red arrow. RBD positive control, produced in HEK293, is present in all anti-RBD blots ranging from 25 to 200 ng; its apparent molecular weight is 35 kDa in reduced conditions. **(B)** Size and tag assessment of the highest-producing clones from the two sets. As denoted in the anti-myc and anti-strep blots, 011_22 is producing a full-sized RBD with both c-myc- and strep- tags.

Expression constructs pNGV010 (with eGFP) and pNGV011, both carrying an RBD modified for ER retention (**Figure 1B**), were first tested in *N. benthamiana* with positive results (data not shown), and then they were used for BY-2 cell transformation. In total, 15 transgenic BY-2 clones accumulated RBD at levels ranging from 45 to 254 µg/g DW (**Figure 5A**). Out of these clones, we selected the best two producers per set of constructs, namely: 010_10, 010_30, 011_2, and 011_22. The selected clones were further analyzed by Western blot for c-myc and strep detection, which revealed that only 010_10 and 011_22 displayed both of the terminal tags (**Figure 5B**).

**Figure 6:**
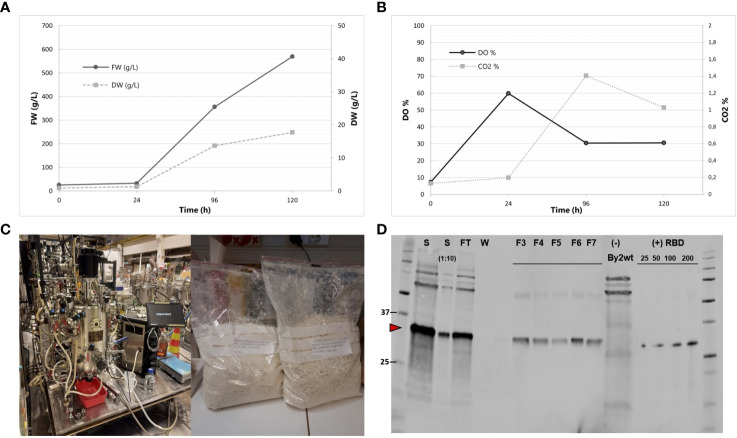
Production of BY2-receptor-binding domain (RBD) in a 20-L bioreactor. **(A)** Fresh weight and dry weight throughout the 5-day cultivation. **(B)** DO% and CO_2_% throughout the 5-day cultivation. **(C)** Biostat C, Sartorius AG 40L Bioreactor and lyophilized biomass resulting from the cultivation. Photos courtesy of Kaisa Rinta-Harri. **(D)** Anti-RBD Western blot of purified BY-2-produced RBD from the bioreactor cultivation. Equal volumes of the samples were loaded to each lane. S (start undiluted and 1:10 dil); FT (flow-through), F3–F7 (fractions 3 to 7); wild-type BY-2 extract as negative control. RBD positive control (SARS-CoV spike protein) is present in a gradient (25, 50, 100, and 200 ng). Its apparent molecular weight is 35 kDa in reduced conditions.

The best RBD-producing BY-2 clone, 011-22, was transferred from calli to liquid cultivation of up to 50 mL volume in shake flasks. The growth behavior of this clone resembled that of wild-type BY-2, peaking at 16–18 g/L DW (**Figures 6A–C**). This selected cell line had a very constant accumulation level of RBD in both the 50-mL shake flask and the 20-L bioreactor scale as the RBD levels were recorded to be 0.26 mg and 0.22 mg RBD/g DW, respectively. The BY-2-produced RBD is referred to as BY2-RBD onwards.

BY2-RBD purification from bioreactor-cultivated biomass via StrepTrap HP chromatography was performed, yielding approximately 8.7 mg of purified RBD from the 20-L batch (**Figure 6D**). The purified BY2-RBD is stable if stored at -80°C in 1× PBS, pH 8, for at least 6 months.

The average time required to manufacture BY2-RBD was estimated to be 14.5 weeks, and in **Figure 4** the total timeframe is reported as broken down into specific activities.

### Analytical characterization of plant-made RBDs

**Figure 7:**
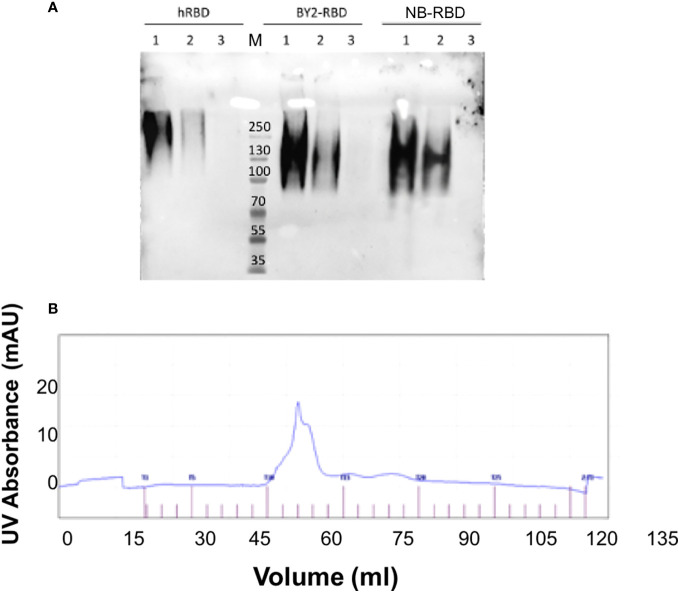
NB-receptor-binding domain (RBD) and BY-2 RBD biochemical comparison. **(A)** Non-reducing PAGE Western blot using the polyclonal anti-RBD PA5-114529 antibody—1: 500 ng, 2: 250 ng, and 3: 50 ng. hRBD: positive control, commercial HEK293-made RBD; BY2-RBD: BY-2-made RBD; NB-RBD: *N. benthamiana*-made RBD. M, molecular marker. **(B)** Output of size exclusion chromatography analysis of NB-RBD; the one of BY2-RBD was identical.

Analytical size exclusion chromatography revealed that both NB-RBD and BY2-RBD eluted in a single peak, suggesting that both RBDs are in a monodispersed phase (**Figure 7A**). When the proteins were further analyzed in a Western blot under non-reducing conditions, the band corresponding to RBD showed the size expected for a tetramer (**Figure 7B**). This was unexpected because the RBD 319-541 displays one single unpaired Cys (Cys538), suggesting that interaction forces other than the disulfide bond promote the formation of RBD tetramers.

### *N*-glycan profiling of plant-made RBDs

**Figure 8:**
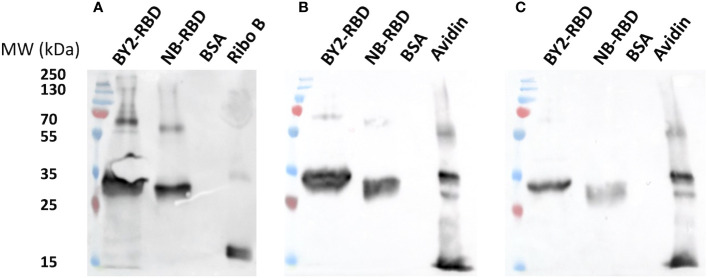
Preliminary N-glycan profiling of NB-receptor-binding domain (RBD) and BY2-RBD via affinodetection with concanavaline A. **(A)** Immunodetection with antibodies raised specifically against core β(1,2)- xylose **(B)** or core α(1,3)-fucose **(C)**. Ribonuclease (Ribo B) and avidine produced in maize (avidine) are positive controls. Bovine serum albumin is a negative control.

A first investigation of the *N*-glycan profiles of NB-RBD and BY2-RBD was performed using western and eastern blot analyses using affinodetection with concanavalin A (Con A), a lectin specific for oligomannosides, and two immunodetections with antibodies raised against core β(1,2)-xylose (anti-xylose antibodies) and α(1,3)-fucose (anti-fucose antibodies), two specific glycoepitopes of plant *N*-glycans (Bardor, 1999). As illustrated in **Figure 8**, both NB-RBD and BY2-RBD were detected with Con A and with anti-xylose and anti-fucose antibodies, demonstrating that recombinant RBD proteins carry both oligomannosides and complex *N*-glycans.

The *N*-glycan profiles of NB-RBD and BY2-RBD were then determined through a glycoproteomic approach as previously described in Balieu et al. (2022). The resulting peptides and glycopeptides were analyzed by nano-liquid chromatography coupled to electrospray mass spectrometry (LC-ESI MS/MS). The overall protein sequence coverages were determined to be about 69% for NB-RBD and 82% for BY2-RBD (**Supplementary Figures S1A, B**). Regarding the protein sequence, a C-terminal peptide having the KDEL extension was identified in the NB-RBD sequence. In contrast, in the BY2-RBD sequence, the C-terminal tryptic peptide (KDEL) generated through proteolysis digestion was too small to be detected by LC-ESI MS/MS. In addition, peptide from F39 and R57 containing the *N*-glycosylation sites was not detected in NB-RBD, suggesting that this peptide is glycosylated. For BY2-RBD, peptide F53-E64 was detected by LC-ESI MS/MS, indicating that this peptide is likely partially *N*-glycosylated.

RBD proteins exhibit two *N*-glycosylation sites located on N42 and 54 for NB-RBD and N55 and 67 for BY2-RBD, corresponding to the same Asn amino acid but displayed in different positions because of the different gene construct used for transformation (**Figures 1A, B**).

In order to determine the respective *N*-glycan profiles of both RBD samples and their distribution on these two *N*‐glycosylation sites, the mixtures of peptides and glycopeptides released by the endoprotease digestions were submitted to a targeted LC-ESI MS/MS analysis (Balieu et al., 2022). To this end, peptides giving MS/MS spectra exhibiting *N*‐glycan diagnostic fragment ions at *m/z* 204 (*N*‐acetylglucosamine) and 366 (Man‐GlcNAc) were selected as being glycopeptides. This allowed the identification of the *N*-glycan structures attached to the two glycosites for the two RBD samples, the graphical description of which is reported in **Supplementary Figure S2**.

**Figure 9:**
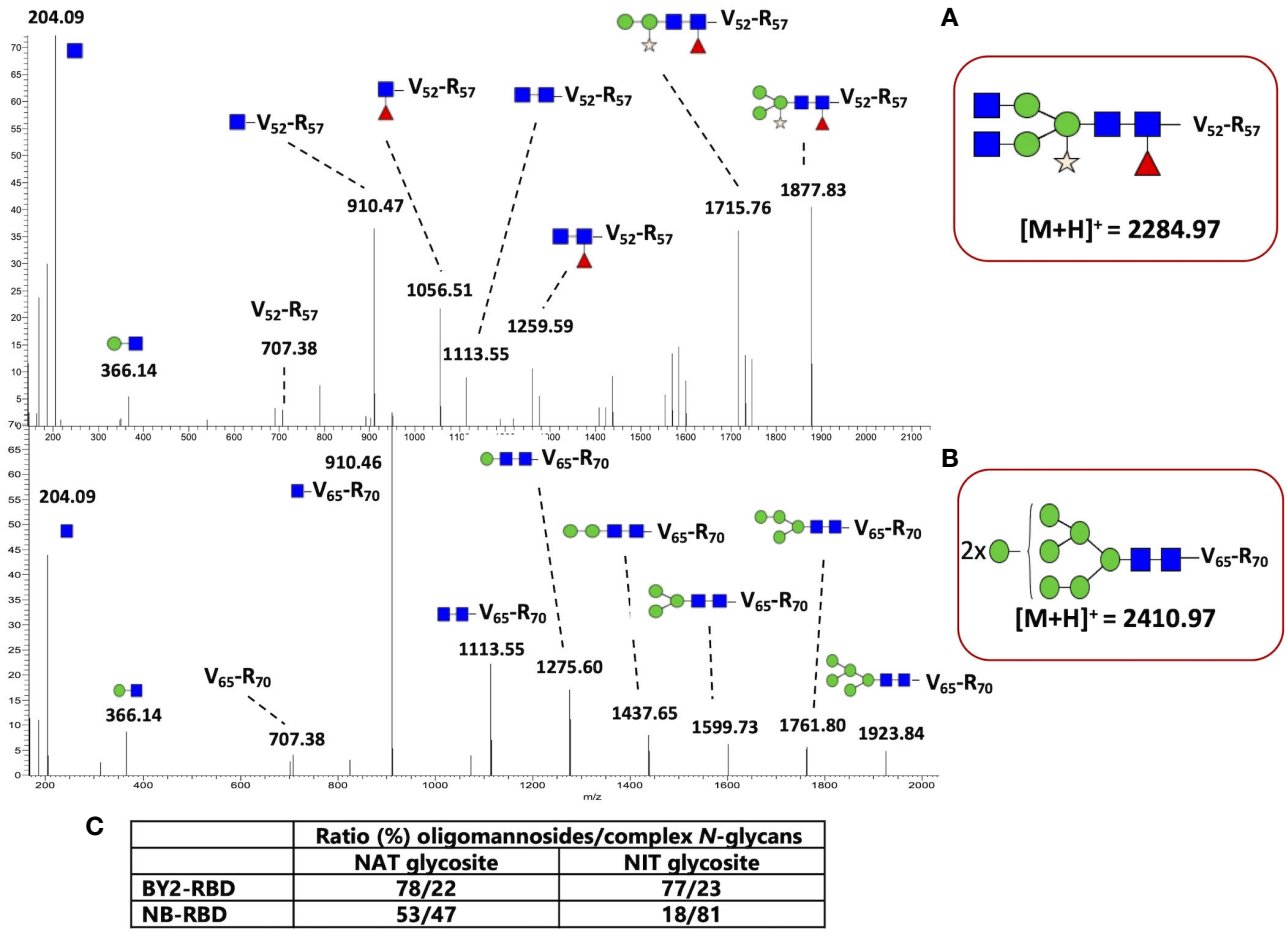
N-glycan profiling of NB-receptor-binding domain (RBD) and BY2-RBD. MS/MS spectra of **(A)** the glycopeptide at *mz* 2,284.97 assigned to the peptide V_52_FNATR_57_ N-linked to Gn_2_M_3_XFGn_2_ from NB-RBD and **(B)** the glycopeptide at *mz* 2,410.97 assigned to the peptide V_65_FNATR_70_ N-linked to Man_8_GlcNAc_2_ from BY2-RBD. GlcNAc, blue square; Man, green circle; Fuc, red triangle; Xyl, yellow star. **(C)** Ratio between oligomannosides and complex N-glycans present on the two specific N-glycosites of RBDs.

The MS/MS spectra of glycopeptides at *mz* 2,284.977 and 2,410.972 were assigned to the peptide VFNATR *N*-linked to either Gn_2_M_3_XFGn_2_ (**Figure 9A**) or to Man_8_GlcNAc_2_ (**Figure 9B**), respectively. In the MS/MS spectrum, in addition to diagnostic ions at 204 and 366, MS/MS ions were assigned to glycopeptide fragments, allowing the determination of glycan sequences.

Numerous MS/MS spectra assigned to glycopeptides were extracted from the generated data, and the glycan sequences of both NB-RBD and BY2-RBD were determined by analysis of the glycan fragment ions. Oligomannosides ranging from Man_5_GlcNAc_2_ to Man_9_GlcNAc_2_ as well as complex glycans with core β(1,2)-xylose and/or α(1,3)-fucose were identified on the two glycosites. In addition, complex *N*-glycans with Lewis glycoepitopes were identified on NB-RBD. Moreover, the relative intensities of each glycopeptide were estimated on the basis of the ion intensities of glycopeptides detected by LC-ESI MS/MS. **Figure 9C** and **Supplementary Figure S3** report on the *N*-glycan distribution on the two glycosites of NB-RBD and BY2-RBD.

### NB-RBD and BY2-RBD present differences in ER retention efficiency

Retention in the ER by a KDEL signal allows the protein to escape the Golgi maturation machinery. As a consequence, KDEL-retained proteins usually exhibit mainly Man_7_GlcNAc_2_ to Man_9_GlcNAc_2_ oligomannosides *N*-linked to their *N*-glycosylation sites (Sriraman et al., 2004; Triguero et al., 2005). The analysis of NB-RBD and BY2-RBD by both western blot and glycoproteomics approaches demonstrated that both oligomannosides and complex *N*-glycans are associated to the two glycosites but with a relative ratio that largely differ between the two recombinant RBDs. **Figure 9C** indicates that retention in the ER was much more efficient in BY2-RBD which presents more oligomannosides (above 77%) than NB-RBD as suggested by its higher ratio of complex *N*-glycans.

### RBD indirect ELISA validation and comparison with EI kit

The comparison between the plant-made RBD-based serological kit with a commercial serological system (Euroimmun) started with the plant-based indirect ELISA absorbance value conversion, performed by the use of a titration curve anti-CR3022 made during indirect ELISA test performance, which resulted in the sera value expression in U/mL (1 U equivalent to 100 ng/mL of monoclonal antibody CR3022; #Ab0168010.0, Absolute Antibody, Oxford, UK) as previously performed in Klausberger et al. (2021).

**Figure 10:**
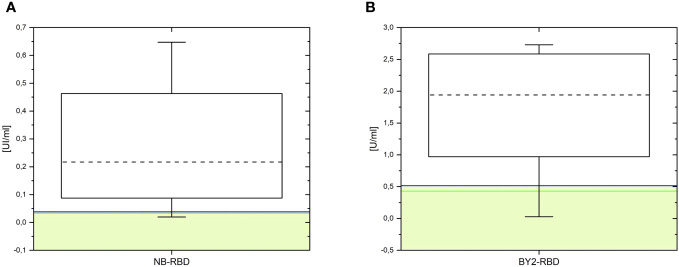
Diagnostic performance of the ELISA tests based on NB-receptor-binding domain (RBD) and BY2-RBD. Box plot distribution (box range, 25–75) of results from ELISA tests based on NB-RBD **(A)** and BY2-RBD **(B)**. Dotted lines mark the medians. The cutoffs are shown in solid lines: the green ones represent the cutoff calculated as the mean plus the double value of SD, while the blue ones represent the cutoff calculated as the mean plus threefold the value of SD.

The diagnostic performances of the two assays were compared in terms of specificity and sensibility, as reported in **Figure 10**, with a set of COVID-19-positive sera (*n* = 20), pre-COVID-19 sera (*n* = 3), and four dilutions of reference antibody. Setting for both plant-based RBD kits a specificity of 100% resulted in a sensitivity of 95% in NB-RBD-based ELISA setting the threshold as the average of healthy sera plus twice their standard deviation and 90% setting the threshold as the average of healthy sera plus three times their standard deviation, while for the BY2-RBD-based ELISA it was 100% in both simulations.

**Figure 11:**
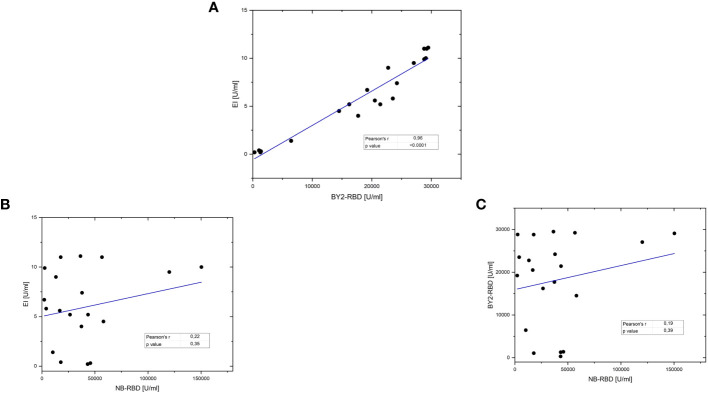
Correlation between ELISA tests based on plant-made receptor-binding domains (RBDs) and the golden standard serological test. Comparison of binding assays by linear regression. The black dots represent the antibody titer in every serum, and the red line represents the trend line. Pearson’s *r* values and relative *p* values are shown in the table on the bottom right of each graph. **(A)** A 20-sera comparison of Euroimmun QuantiVac (EI) with BY2-RBD test. **(B)** A 20-sera comparison of EI with the NB-RBD test. **(C)** A 20-sera comparison of BY2- with NB-RBD tests.

Furthermore, the two assays based on plant-made diagnostic, namely, NB-RBD and BY2-RBD, and the Euroimmune platform were compared. NB-RBD- and BY2-RBD-based ELISA were compared with QuantiVac test with a set of COVID-19-positive sera (*n* = 20), pre-COVID-19 sera (*n* = 3), and four dilutions of reference antibody. We obtained different degrees of correlation with the standard QuantiVac test; a high positive correlation (Pearson’s *r* = 0.96; *p*-value <0.0001) was observed between BY2-RBD ELISA and the standard test, while a lower and not significant correlation was observed between NB-RBD test and the standard one (Pearson’s *r*-value = 0.14 and *p*-value of 0.56) and between NB-RBD- and BY2-RBD-based tests (Pearson’s *r*-value = 0.11; *p*-value of 0.65) (**Figure 11**).

## Discussion

The COVID-19 pandemic prompted the building of a plant scientific hub that easily self-assembled, elicited by the enormous potential of plant science to contribute effectively in fighting present and future pandemics (Avesani and Ponz, 2022). In the recent pandemic scenario, one of the most urgent demands was for diagnostic tests. These tests played a crucial role in identifying infected individuals as the virus outbreak unfolded. As the pandemic progressed, the need for these tests expanded to include estimating the extent of its spread in communities and assessing the effectiveness of vaccination campaigns. Ideally, such tests need to be cost-effective, easy to scale up, rapid to be produced, and reliable.

In the framework of COVID-19, RBD-specific antibody levels correlate well with the induction of functional neutralization responses, which allows monitoring of the dynamics of antibody response after infection and vaccination (Klausberger et al., 2021).

Here we aim to set up a simple enzyme-linked immunosorbent assay (ELISA) format to determine the titers of antibody responses to RBD by manufacturing the recombinant protein in two different plant-based platforms. We then compared their manufacturability in terms of production yields, quality of the purified product, and time needed for the production. Finally, we compared their diagnostic performances in an ELISA serological test with a widely used, high-sensitivity, and high-specificity commercial S1 IgG ELISA kit (Euroimmun).

There are many different host platforms and heterologous expression strategies based on plants, each with specific benefits and drawbacks (Twyman et al., 2003). Plant cell cultures are self-contained systems similar to mammalian cell cultures. They comply with good manufacturing practice (GMP), but on a large scale they are more expensive and elaborate than whole plants. In contrast, leafy crops such as tobacco produce large amounts of biomass and can be scaled up to agricultural levels (Fischer and Emans, 2000; Ma et al., 2003). Transgenic plants have long development timelines, but as permanent genetic resources, they offer batch-to-batch consistency when grown under controlled conditions (Fischer et al., 2012). *Nicotiana* has the potential for transformation into transgenic plants and are also suitable for transient expression, enabling rapid access to material for clinical studies and high yields, but the large-scale use of genetically modified bacteria, in each transformation round, raises the need for more stringent containment and additional labor-intensive procedures (Knödler et al., 2023).

Here we compare the use of stably transformed BY-2 plant cell culture with transiently transformed *N. benthamiana* to produce an ER-retained RBD to setup an ELISA test for serological analysis.

ER retention of RBD was chosen to avoid the attachment on the two available N-glycosylation precursor sites of plant-specific glycans like core xylose and core fucose that could hamper the performance of the diagnostic reagent because of their potential immunoreactivity with human sera (Bardor, 2003). Additionally, ER retention ensured the preservation of the C-terminal tag as previously observed in the production of apoplast-targeted RBD across various plant platforms (Shin et al., 2021; Rebelo et al., 2022).

The *N*-linked glycosylation pathway in plants is well characterized and shares a high degree of homology with other eukaryotic organisms, including site occupancy, frequency of glycosylation, and the structure and composition of the core high-mannose-type glycan added in the ER. Protein *N*-glycosylation starts in the ER with the transfer of the oligosaccharide precursor Glc_3_Man_9_GlcNAc_2_ to specific asparagine (Asn) residues of the polypeptide, followed by limited trimming in both the ER and Golgi and sequential addition of monosaccharides as the proteins travel through the Golgi complex to yield complex *N*-glycans, typically GlcNAc_2_Man_3_XylFucGlcNAc_2_ (GnGnXF) structures (Castilho and Steinkellner, 2012).

The ER-retained RBDs were produced in the two selected plant systems, and both expression systems demonstrated suitability in the production of a functional protein with different yields and timelines for the platform setup. Specifically, comparing the results of this work, the 20-L bioreactor allows us to obtain similar levels of purified RBD (8.7 mg) as 90 4-week-old *N. benthamiana* transiently transformed plants. This quantity may be used to set up 290 ELISA test for the analysis of almost 7,000 sera. The time requested for the production of the diagnostic reagent was estimated to be 14.5 weeks for BY-2 and 6 weeks for *N. benthamiana.* A noteworthy aspect is that both platforms employed for the production of the diagnostic reagent exhibit a linear scalability, emphasizing that the upscale of the process is consistently proportional and predictable.

When it comes to analytical characterization of the plant-made proteins, we detected unexpected difference in the *N-*glycan profiles of the two glycosylation sites present in RBD, with BY2-RBD showing oligo-mannosidic *N*-glycans and NB-RBD displaying a more complex glycan profile, unexpected for an ER-resident protein, thus reflecting the Golgi apparatus maturation of complex *N*-type glycans. We speculate that the observed difference can be explained considering the higher recombinant protein synthesis mediated by MagnICON-vectors that relies upon viral replication components in comparison to a stable expression in BY-2, based on the plant nuclear replication system. The higher transcription rate resulted in higher protein synthesis that may have overloaded the KDEL-mediated retrieval signal to the ER, thus allowing a portion of the recombinant protein to escape by trafficking to the Golgi apparatus as previously shown with other recombinant proteins (Crofts et al., 1999; Navazio et al., 2002).

Consistent with this hypothesis, we observed a degree of toxicity from the RBD expressed in *N. benthamiana* at 3 dpi (not observed when testing BY2-vectors in *N. benthamiana*) that hampered recombinant protein accumulation onwards. We hypothesized that the observed toxicity may reflect the activation at 3 dpi of apoptosis as a consequence of ER overloading, as observed in different systems (Cudna and Dickson, 2003).

The two plant-made proteins were then compared as diagnostic reagents for the setup of an ELISA for the quantification of antibodies present in human sera. Our results suggest that the two plant-made reagents perform differently; when compared in terms of sensitivity and specificity, BY2-RBD-based ELISA allows reaching higher levels of sensitivity than the one based on the use of NB-RBD. Furthermore, when the two tests were used for IgG quantification, the ELISA based on BY2-RBD showed higher correlation levels with the commercial Euroimmun kit than the one based on NB-RBD. We hypothesized that such a difference may be explained by the different glycan profiles of the two recombinant proteins, considering that NB-RBD also bears complex and plant-specific glycans. Complex glycans may be recognized by human sera, but the prevalence of antibodies targeting these types of glycans is rather low.

In the healthy population, Rup et al. (2017) showed, using a validated method, that 13.5% of the subjects were positive to the presence of anti-plant glycan antibodies; these data were consistent with other publications which showed that less than 20% of the normal healthy population have detectable levels of pre-existing antibodies to plant glycan motifs (Landry et al., 2010). Furthermore, complex glycans present on NB-RBD may impact the diagnostic performance of the protein by shielding epitopes or by influencing protein folding, thus modifying protein immunoreactivity in both cases (Watanabe et al., 2020).

Therefore, we speculate that the architecture of complex plant glycans found on NB-RBD may play a role, either directly or indirectly, contributing to the observed reduced correlation in antibody measurements when compared to the current gold standard for antibody assessment, as opposed to BY2-RBD which predominantly features oligomannoside.

It is worth noting that RBD, used as target antigen in our ELISA design, is a portion of the S1 domain of the spike protein used as target antigen in the commercial assay used for comparison; however, previous works demonstrate that such different ELISA design does not significantly impact the accuracy or concordance of serology tests (Thomas et al., 2021).

Unfortunately, in our comparison, we were unable to incorporate the international reference WHO standard (Gundlapalli et al., 2021), which is commonly used *post-hoc* as a reference to define a conversion factor of test unit in BAU per milliliter. This limitation arises from the unavailability of the WHO standard at the time of this study. Nevertheless, a prior study that conducted similar comparisons showed that the use of BAU did not alter the observed levels of correlation (Perkmann et al., 2021).

Overall, our findings demonstrate that plant-made diagnostic reagents can be easily produced by plant systems at yields that can easily cope with emergency scenarios. However, the use of transient expression systems that expedite protein production is associated with increased heterogenicity in the recombinant forms. We hypothesize that this heterogeneity may be attributed to complex plant glycans concealing epitopes on the RBD surface and should be thoroughly examined when setting up a serological test.

## Data availability statement

The original contributions presented in the study are included in the article/**Supplementary Material**. Further inquiries can be directed to the corresponding author.

## Ethics statement

Based on the current regulation on spared serum samples from the diagnostic workup, considering the venous blood sampling as part of the routine clinical practice and the observational nature of the study carried out without any action on the patients themselves, a formal approval by the ethical committee or a signed informed consent was not required. The studies were conducted in accordance with the local legislation and institutional requirements. The human samples used in this study were acquired from a byproduct of routine care or industry. Written informed consent to participate in this study was not required from the participants or the participants’ legal guardians/next of kin in accordance with the national legislation and the institutional requirements.

## Author contributions

MS: Investigation, Writing – original draft. NG-V: Investigation, Writing – original draft. DP: Investigation, Writing – original draft. EZ: Formal analysis, Writing – original draft. ARo: Supervision, Writing – original draft. GS-M: Investigation, Writing – original draft. JB: Investigation, Writing – original draft. PL: Investigation, Writing – original draft. MB: Supervision, Writing – original draft. RC: Investigation, Methodology, Writing – original draft. MC: Investigation, Methodology, Writing – original draft. AM: Supervision, Writing – original draft. ARi: Supervision, Writing – original draft. LA: Conceptualization, Investigation, Project administration, Resources, Supervision, Writing – original draft, Writing – review & editing.

## Supplementary material

Supplementary Figure 1 Sequence coverage determined by LC-ESI MS/MS analysis of **(A)** NB-RBD and **(B)** BY2-RBD. For NB-RBD, peptides from F39 to R57 were not detected, suggesting that they are glycosylated. For BY2-RBD, peptide F53-E64 was detected by LC-ESI MS/MS, suggesting that this peptide is partially N-glycosylated.

Supplementary Figure 2 List of glycans and nomenclature used in the glycoanalysis section of the article.

Supplementary Figure 3 Detailed distribution of the *N*-glycans on the two specific *N*-glycosites of NB-receptor-binding domain (RBD) and BY2-RBD.
