# Supplementary material for: Performance of plant-produced RBDs as SARS-CoV-2 diagnostic reagents: a tale of two plant platforms
Source: Front Plant Sci. 2024 Jan 4;14:1325162. doi: 10.3389/fpls.2023.1325162 (PMC10794598; doi:10.3389/fpls.2023.1325162)
Supplement: Supplementary Figure 1 — Sequence coverage determined by LC-ESI MS/MS analysis of (A) NB-RBD and (B) BY2-RBD. For NB-RBD, peptides from F39 to R57 were not detected, suggesting that they are glycosylated. For BY2-RBD, peptide F53-E64 was detected by LC-ESI MS/MS, suggesting that this peptide is partially N-glycosylated. [file Presentation_1.pptx]

## Slide 1
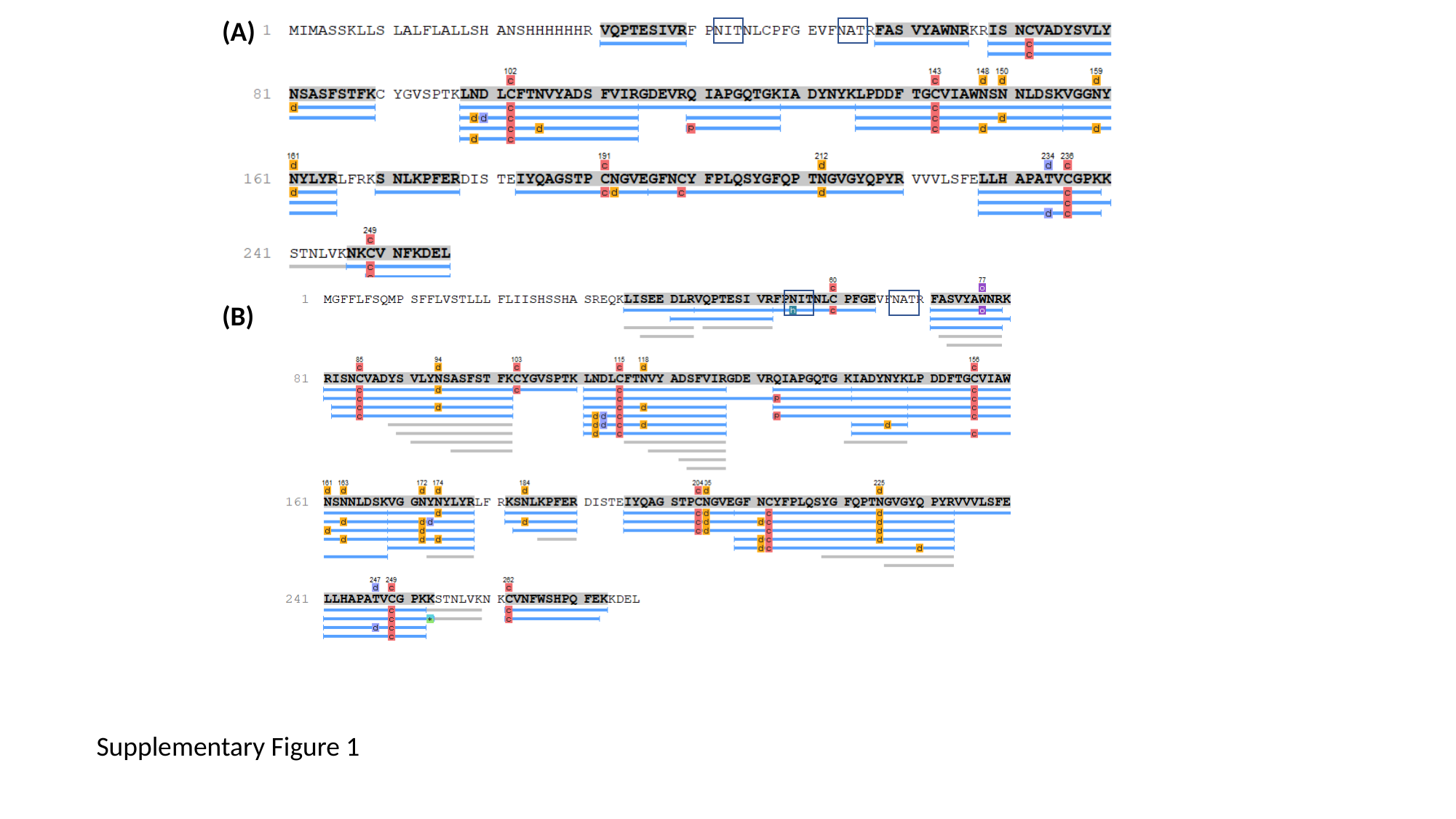

(A)
(B)
Supplementary Figure 1

## Slide 2
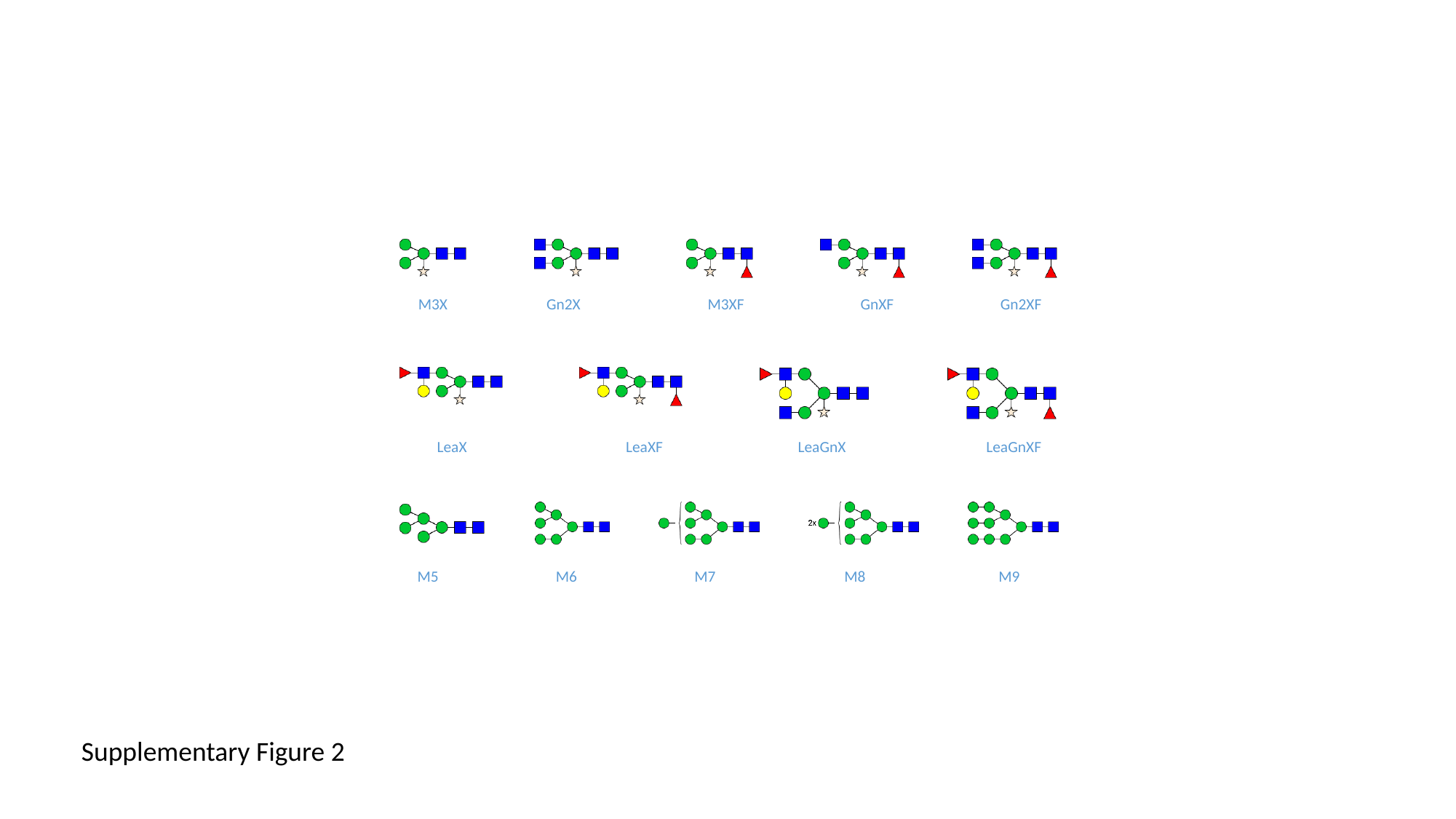

Supplementary Figure 2

## Slide 3
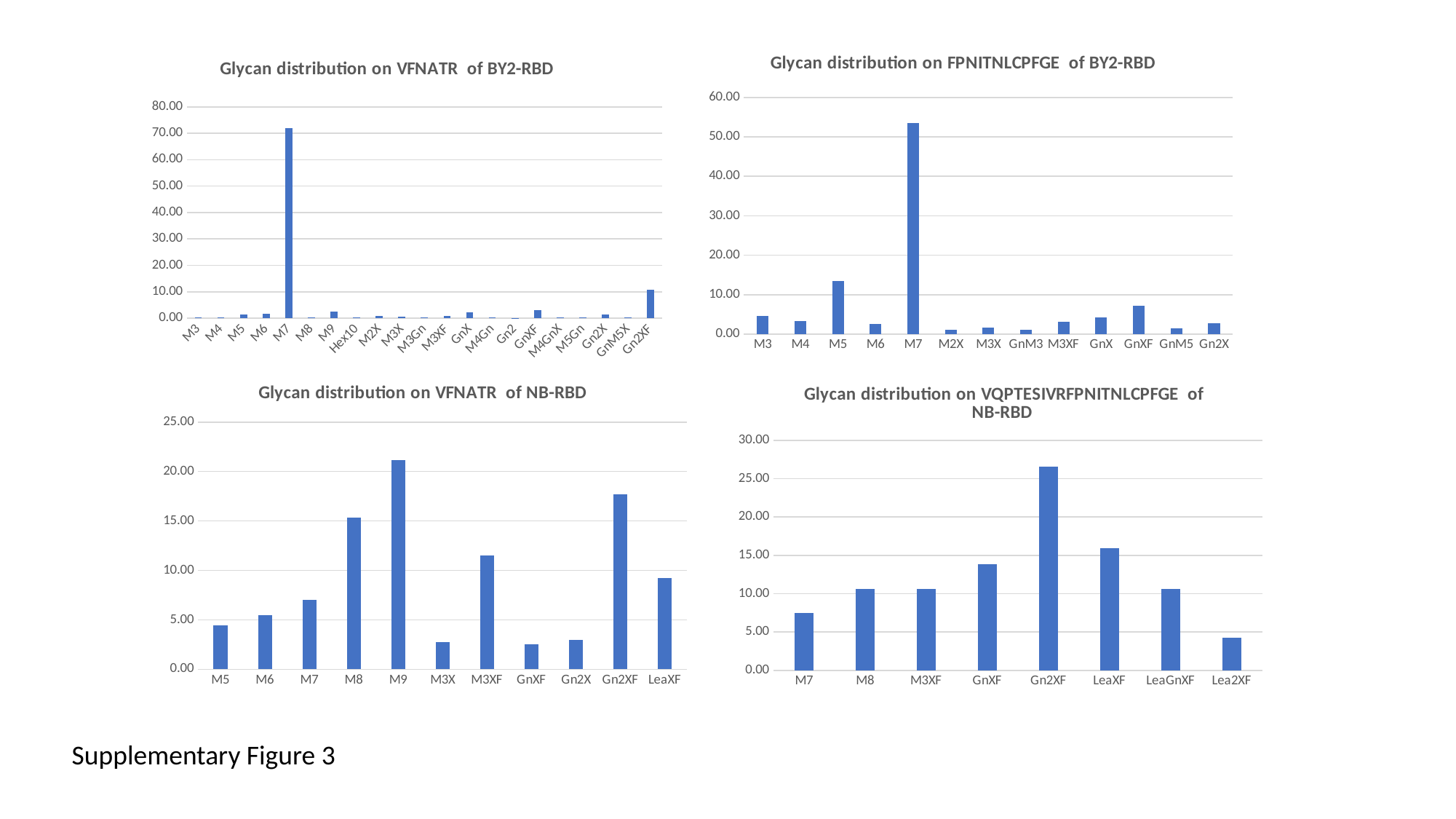

### Chart: Glycan distribution on VFNATR of BY2-RBD
| Category | Pourcentage intensité relative |
|---|---|
| M3 | 0.15064935064935067 |
| M4 | 0.40454545454545454 |
| M5 | 1.2857142857142856 |
| M6 | 1.7337662337662336 |
| M7 | 72.07792207792207 |
| M8 | 0.39935064935064934 |
| M9 | 2.5844155844155843 |
| Hex10 | 0.22142857142857147 |
| M2X | 0.7597402597402597 |
| M3X | 0.5675324675324676 |
| M3Gn | 0.3493506493506494 |
| M3XF | 0.8701298701298701 |
| GnX | 2.305194805194805 |
| M4Gn | 0.15 |
| Gn2 | 0.08831168831168831 |
| GnXF | 3.025974025974026 |
| M4GnX | 0.35000000000000003 |
| M5Gn | 0.16233766233766234 |
| Gn2X | 1.4870129870129871 |
| GnM5X | 0.2889610389610389 |
| Gn2XF | 10.714285714285714 |
### Chart: Glycan distribution on FPNITNLCPFGE of BY2-RBD
| Category | |
|---|---|
| M3 | 4.6796116504854375 |
| M4 | 3.3689320388349513 |
| M5 | 13.398058252427186 |
| M6 | 2.495145631067961 |
| M7 | 53.59223300970874 |
| M2X | 1.145631067961165 |
| M3X | 1.6699029126213591 |
| GnM3 | 1.0970873786407767 |
| M3XF | 3.1844660194174756 |
| GnX | 4.203883495145631 |
| GnXF | 7.213592233009708 |
| GnM5 | 1.5436893203883495 |
| Gn2X | 2.679611650485437 |
### Chart: Glycan distribution on VFNATR of NB-RBD
| Category | |
|---|---|
| M5 | 4.436860068259386 |
| M6 | 5.460750853242321 |
| M7 | 6.996587030716723 |
| M8 | 15.358361774744028 |
| M9 | 21.16040955631399 |
| M3X | 2.7303754266211606 |
| M3XF | 11.49032992036405 |
| GnXF | 2.502844141069397 |
| Gn2X | 2.9579067121729237 |
| Gn2XF | 17.690557451649603 |
| LeaXF | 9.215017064846416 |
### Chart: Glycan distribution on VQPTESIVRFPNITNLCPFGE of NB-RBD
| Category | Pourcentage intensité relative |
|---|---|
| M7 | 7.446808510638299 |
| M8 | 10.638297872340427 |
| M3XF | 10.638297872340427 |
| GnXF | 13.829787234042554 |
| Gn2XF | 26.595744680851062 |
| LeaXF | 15.957446808510639 |
| LeaGnXF | 10.638297872340427 |
| Lea2XF | 4.255319148936171 |Supplementary Figure 3
